# Plant-based vaccines for oral delivery of type 1 diabetes-related autoantigens: Evaluating oral tolerance mechanisms and disease prevention in NOD mice

**DOI:** 10.1038/srep42372

**Published:** 2017-02-13

**Authors:** Amanda L. Posgai, Clive H. Wasserfall, Kwang-Chul Kwon, Henry Daniell, Desmond A. Schatz, Mark A. Atkinson

**Affiliations:** 1 Departments of Pathology, Immunology, and Laboratory Medicine, College of Medicine, University of Florida, Gainesville, FL 32610, USA; 2 Department of Biochemistry School of Dental Medicine, University of Pennsylvania, Philadelphia, PA 19104, USA; 3 Department of Pediatrics, College of Medicine, University of Florida, Gainesville, FL 32610, USA

## Abstract

Autoantigen-specific immunological tolerance represents a central objective for prevention of type 1 diabetes (T1D). Previous studies demonstrated mucosal antigen administration results in expansion of Foxp3^+^ and LAP^+^ regulatory T cells (Tregs), suggesting oral delivery of self-antigens might represent an effective means for modulating autoimmune disease. Early preclinical experiments using the non-obese diabetic (NOD) mouse model reported mucosal administration of T1D-related autoantigens [proinsulin or glutamic acid decarboxylase 65 (GAD)] delayed T1D onset, but published data are conflicting regarding dose, treatment duration, requirement for combinatorial agents, and extent of efficacy. Recently, dogma was challenged in a report demonstrating oral insulin does not prevent T1D in NOD mice, possibly due to antigen digestion prior to mucosal immune exposure. We used transplastomic plants expressing proinsulin and GAD to protect the autoantigens from degradation in an oral vaccine and tested the optimal combination, dose, and treatment duration for the prevention of T1D in NOD mice. Our data suggest oral autoantigen therapy alone does not effectively influence disease incidence or result in antigen-specific tolerance assessed by IL-10 measurement and Treg frequency. A more aggressive approach involving tolerogenic cytokine administration and/or lymphocyte depletion prior to oral antigen-specific immunotherapy will likely be required to impart durable therapeutic efficacy.

In type 1 diabetes (T1D), immunosuppression offered in a variety of forms provides a temporary reprieve from autoimmunity, but is unable to induce lasting immunological tolerance allowing for a reversal of disease once established^1^. The discovery of antigen-specific mucosal tolerance mechanisms (e.g., oral, nasal) has demonstrated utility in preventing autoimmunity^2^,^3^ and as such, precipitated a number of studies in disease-specific animal models^4^,^5^,^6^. Such approaches for inducing tolerance for the reversal and prevention of T1D have been actively pursued both in the non-obese diabetic (NOD) mouse model of the disease as well as in human clinical trials. Indeed, mucosal autoantigen administration has been reported to delay or prevent T1D in NOD mice^7^,^8^,^9^,^10^,^11^. Interestingly, these preclinical studies, while all reporting positive efficacy, are somewhat conflicting regarding the frequency and dosage of antigen administration, species of antigen used, requirement for combinatorial agents and perhaps most importantly, the extent of therapeutic benefit. In humans, nasal insulin administration provided no clinical benefit while imparting subclinical immunological effects^12^. Oral insulin has had limited efficacy, possibly delaying T1D onset in a subpopulation of subjects having high levels of anti-insulin autoantibodies (IAA)^13^,^14^.

A recent report by Pham *et al*. argued that oral insulin treatment is in fact, unable to delay or prevent T1D onset in NOD mice, possibly due to autoantigen digestion prior to reaching the mucosal immune interface, a process that limits bioavailability and subsequent tolerance induction^15^. To address this in the setting of T1D, transgenic plant cells expressing disease-related autoantigens^16^,^17^,^18^,^19^ (i.e., insulin, glutamic acid decarboxylase 65 (GAD)) have been used to deliver intact protein to the small intestine’s gut associated lymphoid tissue. Upon oral delivery, the plant cell wall protects the expressed protein(s) of interest from acids and enzymes in the stomach via bio-encapsulation as human digestive enzymes are incapable of breaking down glycosidic bonds in carbohydrates that make up the plant cell wall. However, when intact plant cells containing autoantigen proteins reach the small intestine, commensal microbes digest the cell wall releasing the autoantigens. When fused with suitable transmucosal carriers (e.g., cholera toxin’s non-toxic subunit B (CTB)), antigens are delivered more efficiently to the immune or circulatory system^20^,^21^,^22^. Transgenes can be expressed in plants via the nuclear or chloroplast genome. Nuclear transgene integration presents fundamental challenges due to low autoantigen expression levels^17^ and risk of transgene dissemination via pollen. As a result, transplastomic tobacco plants have been engineered to express desired proteins in the chloroplast, resulting in consistent high level production of protein due to high copy number of chloroplast genomes while at the same time, eliminating the possibility of unintentional environmental release owing to the maternal inheritance of the chloroplasts^19^,^20^,^23^.

Our goal in this study was to induce autoantigen-specific tolerance and prevent T1D in the NOD mouse via an oral vaccine that could potentially be translated for clinical application. We used transplastomic tobacco expressing two key T1D autoantigens, human proinsulin (hpINS) and GAD. hpINS was expressed as a fusion protein with CTB which binds the monosialotetrahexosylganglioside (GM1) receptor on intestinal epithelial cells and serves as a transmucosal carrier to increase autoantigen bioavailability^19^,^21^. CTB-hpINS ─ GM1 binding has been previously characterized *in vitro*^19^, and CTB-mediated delivery has been validated *in vivo* using tobacco leaves expressing CTB fused to green fluorescent protein^21^. Oral treatment with plant cells expressing CTB-hpINS tobacco was shown to reduce insulitis in NOD mice at 15 weeks of age, but animals were not followed for disease prevention^19^. In similar experiments, oral delivery of plant cells expressing coagulation factors 8 and 9 (FVIII and FIX, respectively) prevented anaphylaxis in murine models of hemophilia by inducing antigen-specific tolerance to those proteins^24^,^25^. Extensive studies on the mechanism of tolerance induction using bioencapsulated CTB-FIX or CTB-FVIII suggest a complex, IL-10 dependent mechanism, ultimately resulting in the induction of CD4^+^CD25^+^Foxp3^+^ Tregs and LAP^+^CD4^+^CD25^−^Foxp3^−^ Tregs that actively suppress anti-FIX or FVIII antibody formation^25^,^26^. Indeed, both CD4^+^LAP^+^ and CD8^+^LAP^+^ cells have been shown to confer tolerance and suppress autoimmune disease^27^,^28^. Moreover even at low doses (1.5 μg), oral administration of the plant-made CTB-acid alpha-glucosidase (GAA) fusion protein prior to GAA injection significantly suppressed GAA-specific immunoglobulin formation in a mouse model of Pompe disease^29^.

In order to improve upon past attempts to prevent T1D via mucosal tolerance and to address unanswered questions regarding dosing and the potential synergistic benefits of combination therapy, we conducted two phases of experimentation. The purpose of the first phase of study was dose optimization. Two transplastomic plants expressing T1D-related autoantigens were each administered at three doses: 25 μg (low), 250 μg (medium), and 500 μg (high) of CTB-hpINS or GAD, once per week for 10 weeks to female NOD mice beginning at 5 weeks of age. Oral treatments were given alone and in combination with 100 μg all-trans retinoic acid (ATRA), which was expected to facilitate mucosal tolerance induction due to its role in TGF-β-mediated induction of Foxp3 expression in T cells^30^,^31^,^32^. In the second phase, we sought to test the optimal dose of each plant material, as determined in phase 1, combine the therapies, and elucidate their potential mechanisms in preventing the disease. The overall aim of this work was to optimize a combination oral therapy to induce autoantigen-specific immunological tolerance and prevent T1D in NOD mice using plants expressing two key autoantigens; however, oral treatments of varying dose, duration, and combination were unable to significantly prevent T1D onset in NOD mice.

## Methods

### Creation of Transplastomic Plants Expressing CTB-hpINS and GAD, and Confirmation of Homoplasmic Lines

Transplastomic tobacco plant leaves expressing CTB-hpINS were created as published^19^. Human GAD65 cDNA was purchased (American Type Culture Collection, Manassas, VA), and the amplified PCR product was cloned into the chloroplast transformation vector, pLD-utr. Plants were transformed using biolistic particle delivery system, and specific integration of the GAD65 expression cassette was investigated as previously described^33^. Total genomic DNA was digested with restriction enzyme, ApaI, and the blotted membrane was probed with radioisotope-labeled flanking region fragment for Southern blot analysis.

### Western Blot Analysis and Quantification of GAD by ELISA

Protein extraction, western blot, densitometric analysis of CTB-hpINS expression, and ELISA for GAD expression were performed as described previously^19^,^34^. Briefly, to extract total soluble proteins, leaves were ground in liquid nitrogen to fine powders; then 100 mg was resuspended in 300 μL extraction buffer [100 mM NaCl, 10 mM EDTA, 200 mM Tris-Cl pH 8.0, 0.1% (v/v) Triton X-100, 400 mM sucrose, 2 mM PMSF, and proteinase inhibitor cocktail] with vigorous vortexing (~30 seconds) followed by sonication using a Misonix Sonicator 3000 (twice pulse on for 5 seconds and pulse off for 10 seconds). The soluble fraction was obtained by centrifuging the homogenate at 13,000 g at 4 °C for 10 minutes. For 10% SDS PAGE, 15 μg soluble proteins were combined with 1X Laemmli sample buffer included with 100 mM DTT and 2% SDS, and boiled for 3 minutes. To detect GAD65 expressed in tobacco leaves by western blot, we applied goat anti-human GAD65 polyclonal primary antibody (Abcam, Cambridge, MA) diluted 1 in 1,500 and donkey anti-goat IgG-HRP detection antibody (Santa Cruz Biotechnology, Dallas, TX) diluted 1 in 3,000 in 3% non-fat dry milk in PBST (0.1% tween 20) solution. Glutathione S-transferase (GST)-tagged GAD2 (molecular weight = 91.8 kDa) was included as a positive control, and untransformed (UT) WT tobacco extracts were included as for a negative control.

### NOD Mice

4 week-old female NOD/ShiLtJ mice were purchased from the Jackson Laboratory (Bar Harbor, ME) and housed 5 mice per cage in the Animal Care Services Biomedical Sciences Building SPF facility at the University of Florida, with food and water available *ad libitum*. All procedures were performed in accordance with guidelines and regulations put forth by the University of Florida Institutional Animal Care and Use Committee (UF IACUC) and according to a protocol approved by UF IACU.

### Autoantigen Preparation and Delivery

#### Phase 1

For phase 1 studies, animals were allocated into 14 treatment groups: three doses of each autoantigen-expressing tobacco leaf material— CTB-hpINS or GAD (25 μg, 250 μg, and 500 μg autoantigen protein), and WT control— each with or without 100 μg ATRA (Sigma-Aldrich Co. LLC; St. Louis, MO) dissolved in tocopherol-stripped corn oil (Fisher Scientific; Pittsburgh, PA). Aliquots were prepared for frozen powdered plant cells: 2.5 g CTB-hpINS plant cells (5 mg CTB-hpINS/g plant cells yielded a total of 12.5 mg CTB-hpINS per aliquot) or 4 g GAD (3 mgGAD/g plant cells yielded a total of 12 mg GAD per aliquot). Frozen powdered plant cells were then, suspended in PBS (final volume of 2.5 mL CTB-hpINS or 2.4 mL GAD) for the “high” dose (500 μg autoantigen/100 μL). The suspension was homogenized for one minute on ice using an Omni GLH (Omni International; Kennesaw, GA), and serial dilutions were performed for the “medium” 250 μg and “low” 25 μg doses. ATRA was dissolved in tocopherol-stripped corn oil at 2 mg/mL. Immediately prior to oral delivery, 300 μL corn oil (with or without ATRA) was emulsified with 600 μL homogenized plant cells between two sterile 3 mL syringes using an emulsification needle. 150 μL of the emulsion was delivered to each mouse via oral gavage. Beginning at 5 weeks of age, the tobacco-oil emulsion was delivered via oral gavage once per week for ten weeks. Mice were followed until T1D onset (treatment failure) or 32 weeks of age.

#### Phase 2

For phase 2 efforts, animals were allocated into 5 treatment groups: 1) Untreated control; 2) WT control tobacco; 3) 500 μg GAD; 4) 250 μg CTB-hpINS; and 5) 500 μg GAD + 250 μg CTB-hpINS. WT and CTB-hpINS plant materials were provided in frozen form (same batch as phase 1) while GAD tobacco materials were in lyophilized form. Plant materials were suspended in PBS and homogenized similar to the methods described above to yield the desired autoantigen doses. The homogenate was not emulsified with oil as ATRA was excluded. 150 μL homogenate was delivered via oral gavage once per week with the combination therapy group receiving two gavages per week (one containing CTB-hpINS and one containing GAD). Weekly oral treatment began at 5 weeks of age and continued until animals reached one of the defined endpoints: cross-sectional time points − 6, 10, and 14 weeks of age (n = 3 per group) or longitudinal time points - T1D onset or 32 weeks of age (n = 9–11 per group).

### Progression to Diabetes

Beginning at 10 weeks of age, blood glucose was measured once per week via tail prick using an AlphaTRAK2 (Abbott Animal Health; Abbott Park, IL) glucose meter. Animals with blood glucose values (BGVs) ≥ 250 mg/dL were rescreened the following day. Diabetes was defined as two consecutive BGVs ≥ 250 mg/dL within 24 hours.

### Necropsy and Processing of Tissues

At the defined endpoints, mice were sacrificed and blood, spleen, pancreas, and when possible, mesenteric and pancreatic lymph nodes (MLN and PLN, respectively) harvested. Serum was stored at −20 °C. Spleens, MLN, and PLN were freshly processed to single cell suspensions and red blood cells lysed as previously described^35^. Pancreata were fixed in 10% neutral buffered formalin at room temperature overnight, transferred to PBS, and stored at 4 °C.

### Flow Cytometry

10^6^ spleen, MLN, and PLN cells were stained as described^35^ for flow cytometric analysis [CD8b-FITC (clone eBioH35–17.2), CD4-PE Cyanine7 (clone GK1.5), CD25-APC (clone PC61.5), Foxp3-PE (clone NRRF-30), and in phase 2, LIVE/DEAD^®^ Fixable Near-IR Dead Cell Stain Kit (Life Technologies; Grand Island, NY), LAP-PerCP eFluor710 (clone TW7-16B4)] using a BD LSRFortessa (Franklin Lakes, NJ) and FCS Express 4 Flow Research Edition software (De Novo Software; Glendale, CA). Lymphocytes were gated on forward and side scatter. Dead lymphocytes staining strongly positive for LIVE/DEAD^®^ Fixable Near-IR were excluded. Antibodies were purchased from eBioscience (San Diego, CA).

### ELISPOT

#### Phase 2

Splenocytes were analyzed for IFN-γ and IL-10 production in response to antigen-specific stimulation via ELISPOT kits (BD; Franklin Lakes, NJ) according to manufacturer’s instructions. Briefly, cells were plated in triplicate at 3 × 10^5^ cells/well with the following stimulatory conditions: 1) unstimulated; 2) recombinant human insulin (1 μg/ml) (Sigma-Aldrich Co. LLC; St. Louis, MO); 3) recombinant human GAD (1 μg/ml) (KRONUS Inc.; Star, ID); 4) recombinant CTB (1 μg/ml) (Sigma-Aldrich Co.); and 5) soluble anti-CD3 (5 μg/ml) + anti-CD28 (2.5 μg/ml) (eBioscience). Cells were incubated at 37 °C and 5% CO_2_ for 65 hours. Cytokine spots were developed (6 minutes for IFN-γ and 15 minutes for IL-10) using 3-amino-9-ethylcarbazole (AEC) substrate (BD) according to manufacturer’s instructions. Spots were enumerated using a Bioreader^®^ 4000 Pro-X and the Bioreader^®^ software generation 8 (BIOSYS USA LLC; Miami, FL). Spots per well were summed, averaged across triplicates, and reported as a stimulation index relative to unstimulated cells.

### Luminex

Sera collected at 4 and 16 weeks of age were diluted 1:12,500 and analyzed for total IgA, IgM, IgG1, IgG2a, and IgG2b titers via Milliplex^®^ MAP Mouse Immunoglobulin Magnetic Bead Panel─Isotyping Multiplex Assay kit according to manufacturer’s filter plate procedure instructions (EMD Millipore; Billerica, MA). Plates were run on a Luminex^®^ 200 Multiplexing Instrument with xPONENT^®^ software version 3.1 (Luminex; Austin, TX) and analyzed using Milliplex^®^ Analyst software version 5.1.0 (EMD Millipore; Billerica, MA).

### ELISA

Sera collected at 4, 8, and 12 weeks of age were analyzed for insulin- and CTB-specific IgG, IgA, and/or IgM titers via ELISA. All antibodies were purchased from Abcam^®^ except anti-mouse IgA detection antibody which came from ELISA Ready-Set-Go!^®^ kit (eBioscience, Inc.; San Diego, CA). High binding clear 96-well microplates (Greiner Bio-One; Monroe, NC) were coated with 50 μL/well recombinant human insulin (Sigma-Aldrich Co.) or CTB (Sigma-Aldrich Co.) at 5 μg/mL in 0.1 M NaHCO_3_ (pH 9) overnight (O/N) at 4 °C and then, blocked with 200 μL/well 10% FBS O/N at 4 °C. For IgG, anti-insulin and anti-CTB standard curves were generated using mouse anti-insulin IgG clone IN-05 and mouse monoclonal anti-CTB IgG diluted in 10% FBS. Sera were diluted 1:30 in 10% FBS and added at 50 μL/well to duplicate wells. Plates were incubated O/N at 4 °C and washed five times with PBS containing 0.05% Tween 20. HRP-conjugated detection antibodies - polyclonal goat anti-mouse IgG-Fc, anti-mouse IgA detection antibody, and rat anti-mouse IgM-mu chain clone SB73a - were diluted in 10% FBS (1:40,000 IgG, 1:500 IgA, and 1:3,000 IgM), added at 50 μL/well, and incubated for 1 hour at RT. Plates were washed as described above. 50 μL/well TMB (eBioscience, Inc.; San Diego, CA) was added and developed at RT in the dark for 15 minutes. The reaction was stopped with 50 μL/well 1 N HCl (Fisher Scientific). Optical density was read immediately at 450 nm on a SpectraMax M5 microplate reader with SoftMax^®^ Pro 4.8 Data Acquisition and Analysis Software (Molecular Devices, LLC.; Sunnyvale, CA).

### Histology

Fixed, paraffin-embedded pancreata were stained via IHC at the University of Florida Molecular Pathology Core lab and evaluated for immune-phenotyping of the insulitic lesion. One section/pancreas was stained for CD3 (peroxidase-DAB) and B220 (alkaline phosphatase-Fast Red) and counterstained with hematoxylin as previously described^36^. Stained slides were scanned using an Aperio ScanScope CS and Spectrum Plus version 11 at 20 × magnification (Aperio Technologies; Vista, CA). Images were annotated using the Aperio ScanScope viewing program to exclude exocrine tissue. An area quantification macro was optimized, and analysis was completed using for the CytoNuclear IHC quantification software (Indica Laboratories; Albuquerque, NM) to enumerate islet area occupied by CD3^+^ T cells and B220^+^ B cells. Pancreata were flattened prior to processing, allowing for examination of the entire tissue, with an average number of islets examined per section = 31.84 ± 1.805 (Mean ± standard error of the mean (SEM)).

### Statistical Methods

Data analyses were conducted using GraphPad Prism software v5.01 (GraphPad Software, Inc.; La Jolla, CA) for one-way ANOVA, two-way ANOVA, unpaired student’s t-test, chi-square test, and Kaplan-Meier life table analysis. Statistical significance was defined as *P* < 0.05. Error bars represent the mean ± SEM.

## Results

### Creation of Transplastomic Plants Expressing CTB-hpINS and GAD

**Figure 1:**
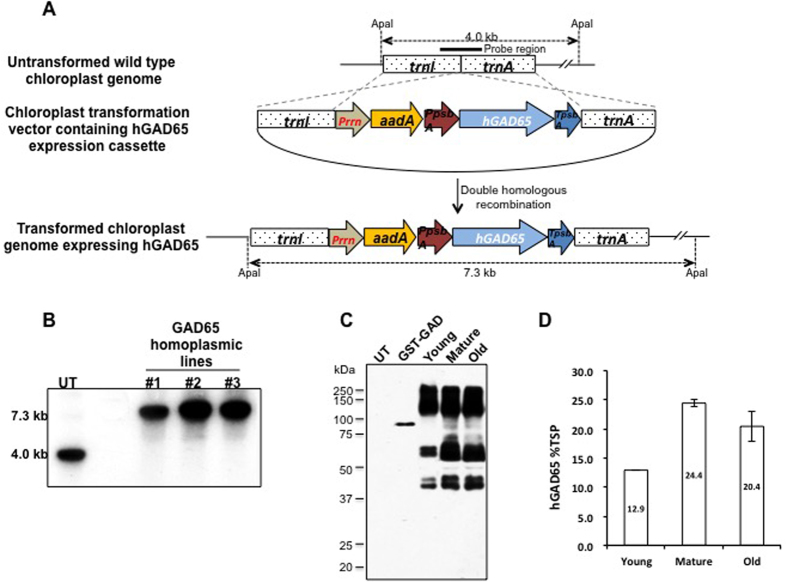
Creation of transplastomic tobacco lines expressing human GAD65 and evaluation of expression. (**A**) Schematic diagram of chloroplast vectors containing the *hGAD65* gene constructed into the chloroplast transformation vector. (*Prrn* = chloroplast rRNA operon promoter; *aadA* = aminoglycoside 3′-adenylytransferase gene; *PpsbA* = *psbA* gene promoter; *hGAD65* = coding sequence of human GAD; *TpsbA* = 3′ UTR of *psbA* gene; *trnI* = isoleucyl-tRNA; *trnA* = alanyl-tRNA). (**B**) Southern blot analysis of ApaI digested total DNA from untransformed control (UT) and GAD transplastomic tobacco lines (#1, #2, and #3) probed with the radioisotope-labeled flanking region fragment as shown in (**A**). (**C**) Western blot probed with GAD antibody; 15 μg of total leaf soluble protein was loaded in each lane; UT: untransformed plant; young, mature, and old transplastomic leaves were harvested at 6 PM. GST-GAD: 100 ng, positive control. ELISA quantitation using goat polyclonal anti-GAD65 (diluted 1:1500), is shown as a percentage of the total soluble proteins (TSP). Data expressed as mean ± SD of three independent experiments.

Transplastomic tobacco plants expressing CTB-hpINS were created as published previously^19^. Plants expressing human GAD were created to test the potential for synergistic benefit in combining two autoantigens to induce oral tolerance and prevent TID in NOD mice. Antigen fusion to CTB has been shown to lower the dose required for oral tolerance induction^17^,^37^. However, attempts to express CTB-GAD in plant chloroplasts were unsuccessful. Therefore, this study was performed only with GAD expressed in chloroplasts and not CTB-GAD. The human GAD (*hGAD65*) gene was inserted into the chloroplast transformation vector – pLDutr. The constructed transformation vector was designed to allow for the specific integration of *hGAD65* expression cassette into the intergenic space between isoleucyl-tRNA synthetase (*trnI*) and alanyl-tRNA synthetase (*trnA*) of the wild type chloroplast genome by double homologous recombination (Fig. 1A). The *hGAD65* gene expression in chloroplasts was driven by the light regulated *psb*A promoter and 5′ untranslated region (UTR) to increase expression, and the expressed transcripts were stabilized by the *psb*A 3′ UTR (Fig. 1A). Transplastomic plants were subject to Southern blot assay to evaluate homoplasmy (transformation of all chloroplast genomes). Three independent lines showed transformed fragments of 7.3 kb while the untransformed wild type control fragment migrated to 4.0 kb (Fig. 1B) when probed with the flanking sequence (Fig. 1A). Absence of the native untransformed chloroplast genome 4.0 kb fragment in transplastomic lines (Fig. 1B) confirmed homoplasmy.

To evaluate GAD expression, leaves of different ages were harvested at 6 pm for maximum yield, and total soluble proteins were extracted and analyzed via western blot. In addition to polypeptides at approximately 65 kDa (correct size), >140 kDa and 40 kDa polypeptides cross-reacting with the GAD antibody were also observed (Fig. 1C), probably representing a cleaved product and post-translational modification of GAD65 within the chloroplasts including phosphorylation, acetylation^38^, and palmitoylation^39^,^40^. Additions of these small molecules via post-translational modification appeared to cause a small increase in molecular weight at the 65 kDa band (Fig. 1C), supporting the notion that lipidation of GAD65 could cause hydrophobic aggregation during the boiling step of sample preparation, resulting in the higher molecular weight bands^41^. However, the cleaved product was not observed in native gels (data not shown), indicating that this may be the artifact of the SDS gel. Primary antibody specificity was confirmed using GST-tagged GAD2 positive control and WT tobacco extracts as a negative control. GAD expression levels were quantified via ELISA. Expression varied between 12.9% and 24.4% of total soluble protein (TSP) and was highest in mature leaves (Fig. 1D). Therefore, the mature leaves harvested at 6 pm were used for animal studies. The average expression levels (mg/g plant powder) were 5.42 mg/g CTB-hpINS (frozen) and 3.33 mg/g GAD (frozen) or 15.3 mg/g GAD (lyophilized).

### Preclinical Effects of Oral Therapies on Diabetes Progression in NOD mice

#### Oral Therapy with Plants Expressing CTB-hpINS or GAD appeared to delay T1D Onset in NOD Mice but this effect was not statistically significant

**Figure 2:**
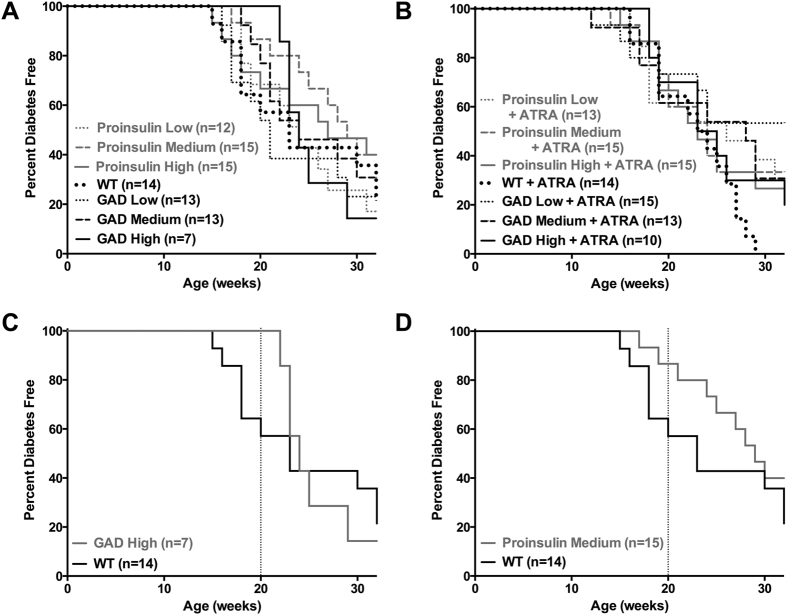
Evaluation of T1D in NOD mice after oral delivery of CTB-hpINS (human proinsulin) or GAD bioencapsulated in plant cells. T1D progression was not significantly different in animals receiving autoantigen-expressing tobacco versus control (WT) tobacco (**A**) without or (**B**) with ATRA (*P* = 0.71 and *P* = 0.47, respectively). (**C,D**) Early T1D onset was defined as occurring prior to 20 weeks of age, indicated by a dashed line. There was a trend toward delayed T1D onset in mice that received oral treatment with (**C**) 500 μg GAD (GAD High) and (**D**) 250 μg CTB-hpINS (Proinsulin Medium; gray lines) versus WT (black line) control tobacco, *P* = 0.58 and *P* = 0.16, respectively, (Gehan-breslow-wilcoxon test).

In the first round of experiments (phase 1), NOD mice received weekly oral gavages for 10 weeks beginning at 5 weeks of age. Gavages containing three doses of GAD, CTB-hpINS or WT control were administered alone as well as in combination with ATRA (Fig. 2A,B). Six weeks after completion of oral feeding (at 20 weeks of age), 57% of NOD mice fed with untransformed leaves were diabetes free, whereas 100% of mice fed with the highest dose of GAD (n = 7, Fig. 2C) and 87% of mice fed with the medium dose of CTB-hpINS (n = 15, Fig. 2D) were diabetes free. Thus, there was a trend (*P* = 0.07) toward reduced frequency of early diabetes onset (prior to 20 weeks of age) in both of these groups compared to animals treated with WT tobacco (Fig. S1). These results are similar to previous studies using the same CTB-hpINS plant materials^19^ even though the amounts of antigen fed in this study are approximately 1.8, 17.9 and 35.7 fold higher for the low, medium, and high doses, respectively. However at 32 weeks of age, there was no significant difference in T1D incidence (Fig. 2). Addition of ATRA did not have any beneficial effect (Fig. 2B).

#### Combination Oral Therapy with CTB-hpINS plus GAD. Did Not Result in Synergism to Prevent T1D Onset in NOD Mice

Since monotherapy appeared to delay early diabetes onset, we sought to determine whether combination therapy would be synergistic for sustained tolerance induction and disease prevention. We also hypothesized that the tolerogenic effect of phase 1 therapy might be lost upon treatment cessation and therefore, continued oral therapy throughout the study’s entirety. We chose the two most effective therapies (i.e., the medium dose of CTB-hpINS and the highest dose of GAD) for use in combination for phase 2 efforts. Beyond this, since the phase 1 experiments demonstrated no therapeutic benefit of combination with ATRA, this was not included during phase 2.

**Figure 3:**
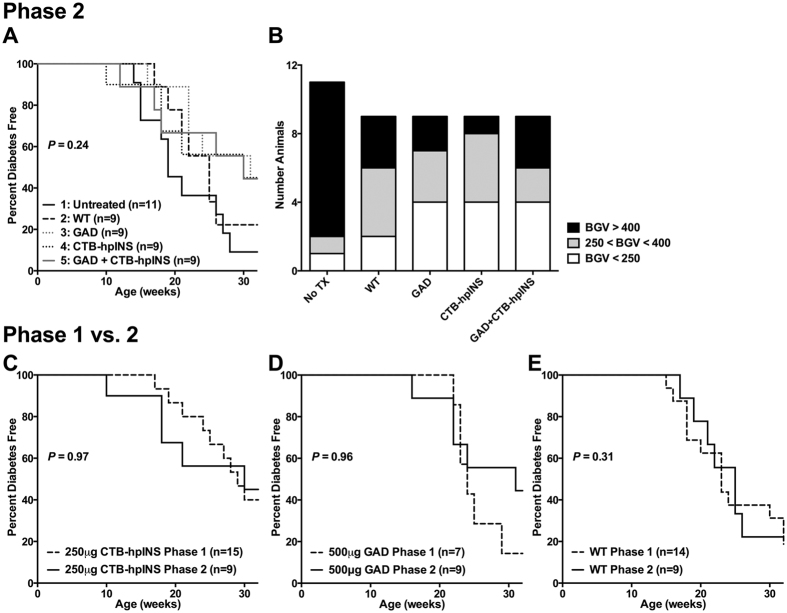
(**A**) Continuous oral treatment with tobacco leaves expressing 500 μg GAD (gray, short dash), 250 μg CTB-hpINS (black, short dash), or the combination of both (gray, solid line), were compared to untreated (black, solid line) or WT control tobacco-treated animals (black, long dash), *P* = 0.24 (Log-rank test). (**B**) Animals experiencing catastrophic failure (black), non-catastrophic failure (gray), or no failure (those animals that survived to 32 weeks of age, white) are represented *P* = 0.08 (Chi-square test). T1D incidence did not differ between Phase 1 (dashed lines, treatment duration: 10 weeks) and Phase 2 (solid lines, treatment duration: ongoing through study endpoint) for (**C**) CTB-hpINS, (**D**) GAD, and (**E**) WT tobacco-treated animals *P* = 0.97, *P* = 0.96, and *P* = 0.32, respectively (Log-rank test). Number of animals (n) per group is indicated in the figure.

There was a 22% difference in the proportion of diabetic mice between those autoantigen-treated and WT-treated animals (Fig. 3A); however, the study was not sufficiently powered to detect a difference less than 50%, an effectiveness in line with previous oral antigen studies in NOD mice. Thus, T1D incidence and progression did not differ significantly between NOD mice that received oral autoantigen therapy (either alone or in combination). Compared to tobacco-treated animals (including WT), untreated mice had a more dramatic onset of diabetes with at least one of the two consecutive diagnostic BGVs exceeding 400 mg/dL in the majority of those animals (Figs 3B, S2A–E). The CTB-hpINS fed group showed the lowest rate of severity, with 11% (n = 1/9) mice showing >400 mg/dL diagnostic BGV (Fig. 3B). In the previously reported study^19^, none of the untreated mice showed >400 mg/dL BGV at any point, though those animals were sacrificed prior to expected time of peak T1D incidence.

Results were highly reproducible between phase 1 and phase 2: 87% in phase 1 (n = 13/15) and 67% in phase 2 (n = 6/9) of animals fed 250 ug CTB-hpINS remained diabetes free at 20 weeks of age with total T1D incidence of 60% (phase 1) and 56% (phase 2) by 32 weeks (Fig. 3C). Similarly, 100% (n = 7) in phase 1 and 89% (n = 8/9) in phase 2 of NOD mice that received 500 ug GAD alone remained T1D free to 20 weeks (Fig. 3B). T1D progression was also comparable between phases 1 and 2 for animals receiving WT control tobacco (Fig. 3E). Continued feeding of either CTB-hpINS or GAD did not sustain the possible beneficial effects observed up to 20 weeks suggesting the need to explore other key factors involved in long-term maintenance of tolerance.

### Immunological Effects of Oral Therapies in NOD mice

#### Prolonged Oral Autoantigen Therapy Increased Tregs in the Spleen but Not Disease-Associated Draining Lymph Nodes

**Figure 4:**
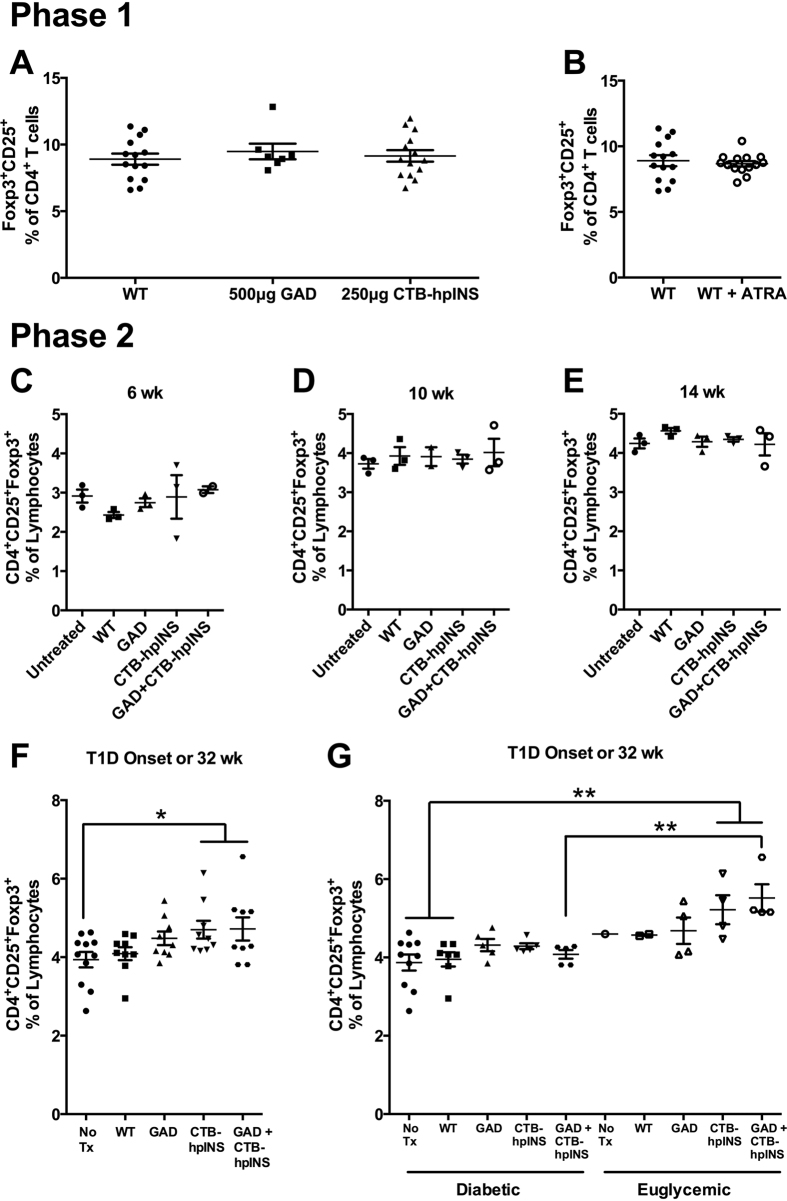
For flow cytometric analysis, cells were gated on lymphocytes and then, on the CD4^+^ population to evaluate CD25^+^Foxp3^+^ Treg frequency in the spleen. (**A**) Phase 1 treatment of NOD mice with tobacco expressing 500 μg GAD (n = 7) or 250 μg CTB-hpINS (n = 14) without ATRA did not affect Treg frequency in the spleen compared to treatment with WT (n = 14), *P* = 0.73. (**B**) Treatment with ATRA + WT tobacco (n = 14) did not affect Treg frequency compared to WT tobacco-treated mice (n = 14), *P* = 0.62 (ANOVA).At (**C**) 6, (**D**) 10, and (**E**) 14 weeks of age as well as (**F,G**) at T1D onset or 32 weeks of age, fresh splenocytes were stained for CD4, CD8, CD25, LAP, and Foxp3 for flow cytometric analysis. Live lymphocytes were analyzed for the percent of CD4^+^CD25^+^Foxp3^+^ Tregs. (**C–E**) There was no difference in Treg frequency at cross-sectional time points, *P* = ns (all). (**F**) At study endpoint (T1D onset or 32 weeks of age), mice treated with CTB-hpINS tobacco, alone or in combination with GAD tobacco, demonstrated significantly greater Treg frequencies compared to untreated NOD mice, *P* < 0.05. (**G**) Among animals that received combination therapy, Treg frequency was significantly higher in those that were euglycemic at 32 weeks of age compared to diabetic animals, *P* < 0.001 (ANOVA). No Tx indicates untreated control NOD mice.

**Figure 5:**
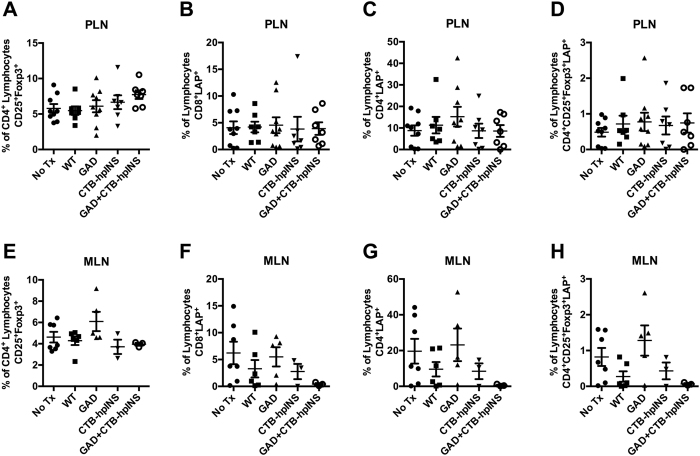
At T1D onset or 32 weeks of age, (**A–D**) PLN and (E-H) MLN cells were stained for CD4, CD8, CD25, LAP, and Foxp3 for flow cytometric analysis. Live lymphocytes gated on CD4 were analyzed for the percent of CD25^+^Foxp3^+^ Tregs. There was no difference in Treg frequency in the (**A**) PLN or (**E**) MLN, *P* = 0.29 and *P* = 0.12, respectively. There was no significant difference in the percent of live lymphocytes that were (**B,F**) CD8^+^LAP^+^ T cells, *P* = 0.997 and *P* = 0.32, respectively; (**C,G**) CD4^+^LAP^+^ T cells, *P* = 0.58 and *P* = 0.23, respectively; and (**D,H**) CD4^+^CD25^+^Foxp3^+^LAP^+^ Tregs, *P* = 0.89 and *P* = 0.06, respectively (ANOVA). No Tx indicates untreated control NOD mice.

In phase 1, there were no observed changes in CD4^+^CD25^+^Foxp3^+^ Treg frequency in the spleens of mice treated with tobacco expressing 500 μg GAD, 250 μg CTB-hpINS, or WT tobacco (Fig. 4A). The inclusion of ATRA was similarly not associated with an increase in Treg frequency in the spleen (Fig. 4B). In phase 2, there were no differences in CD4^+^CD25^+^Foxp3^+^ Treg frequency within the spleen at 6, 10, and 14 weeks of age (Fig. 4C–E). However, splenic Treg frequencies were increased in mice treated with CTB-hpINS as well as CTB-hpINS plus GAD, relative to untreated animals at longitudinal endpoints (Fig. 4F). This effect was particularly evident among treated mice that did not progress to hyperglycemia (Fig. 4G). LAP^+^ T cells were not detectable in the spleen for any time point or treatment group. There were no changes in the frequency of CD4^+^CD25^+^Foxp3^+^ Tregs or LAP^+^ T cells in the PLN or MLN in any animals – progressors or not (Fig. 5 and S3).

#### Oral Autoantigen Therapy Did Not Induce Antigen-Specific Tolerance

Oral autoantigen therapy failed to induce antigen-specific tolerance as measured by ELISPOT for splenocyte IL-10 and IFN-γ production in response to insulin, GAD, or CTB stimulation (Fig. S4). While IFN-γ production was reduced in response to insulin stimulation in CTB-hpINS-treated animals at 14 weeks of age (Fig. S4I), there was no increase in IL-10 production (Fig. S4K). However, these responses were not sustained at longitudinal endpoints (T1D onset or 32 weeks of age) (Fig. S4M,O). In fact, at these time points, splenocytes from animals treated with CTB-hpINS demonstrated reduced IL-10 production in response to GAD stimulation compared to splenocytes from untreated controls, which may indicate an unexpected off-target side-effect and failure to induce bystander suppression (Fig. S4P).

#### Oral Therapy Induced Differential Effects on Humoral Immunity

**Figure 6:**
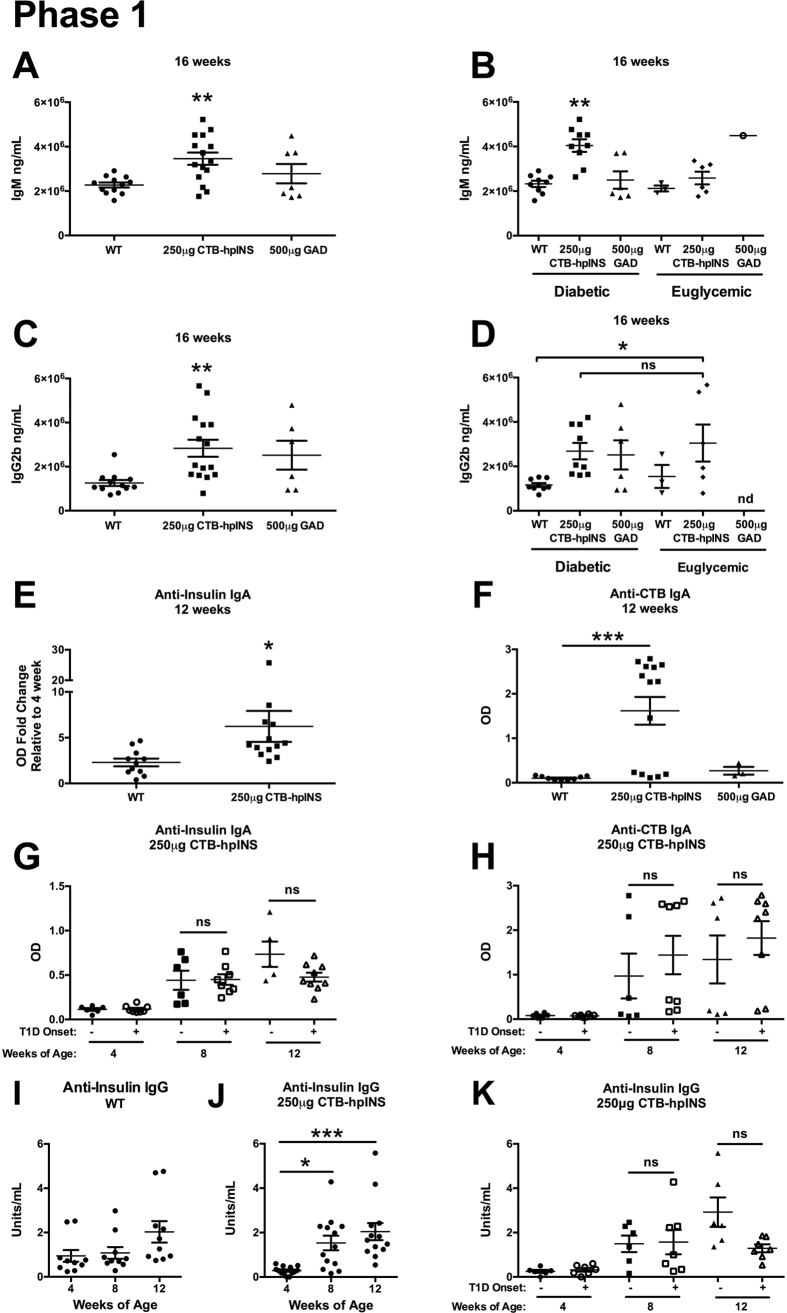
Phase 1: At 16 weeks of age, total serum (**A**) IgM and (**C**) IgG2b were increased in animals treated with tobacco expressing 250 μg CTB-hpINS compared to WT-treated animals, *P* < 0.01 and *P* < 0.01, respectively. (**B**) High IgM was unique to animals that would progress to T1D, *P* < 0.0001. (**D**) Total serum IgG2b was higher in euglycemic animals that received 250 μg CTB-hpINS compared to diabetic controls but not compared to treated animals that would become diabetic, *P* < 0.05 (ANOVA). (**E**) Anti-insulin IgA, as measured by OD fold change relative to 4 weeks of age, was significantly different between WT and CTB-hpINS-treated animals at 12 weeks of age, *P* < 0.05 (unpaired student’s t-test). (**F**) Anti-CTB IgA as measured by absolute OD was significantly increased in animals treated with tobacco expressing 250 μg CTB-hpINS compared to WT, *P* < 0.001 (ANOVA). Among animals treated with plant leaves expressing CTB-hpINS, (**G**) anti-insulin and (**H**) anti-CTB IgA levels did not differ between animals that would remain euglycemic (filled shapes) or become diabetic (open shapes) during the course of the study, *P* < 0.0001 and *P* < 0.001, respectively (ANOVA). (**I**) Anti-insulin IgG titers did not differ significantly over time in animals treated with WT tobacco, *P* = 0.08. (**J**) Animals treated with 250 μg CTB-hpINS displayed increased anti-insulin IgG at 8 and 12 weeks, relative to 4 weeks of age, *P* < 0.001 (ANOVA), but there was no difference in directly comparing WT- and CTB-hpINS treated animals at 12 weeks of age, *P* = 0.99 (unpaired student’s t-test). (**K**) Anti-insulin IgG titers did not differ between animals that would become diabetic or remain euglycemic during the study, *P* < 0.001 (ANOVA). No Tx indicates untreated control NOD mice.

In phase 1, total serum IgM, IgG1, IgG2b, or IgG3 levels did not differ between treatment groups prior to the initiation of oral treatment (Fig. S5A–D). At 16 weeks of age, total IgG1, IgG3, and IgA did not differ across treatment groups (Fig. S5E–G) or between progressors and non-progressors to T1D (Fig. S5H–J), but total serum IgM was elevated in the 250 μg CTB-hpINS-treated animals (Fig. 6A) that progressed to diabetes (Fig. 6B). However, the effect was not antigen-specific (for insulin or CTB) (Fig. S5K–L). Similarly, total IgG2b was increased in mice that received CTB-hpINS compared to WT-treated animals at 16 weeks (Fig. 6C). Because IgG2b class switching is induced by TGF-β, it has been used as a marker for mucosal tolerance^42^,^43^,^44^, but IgG2b levels did not associate with disease onset among CTB-hpINS-treated animals (Fig. 6D).

**Figure 7:**
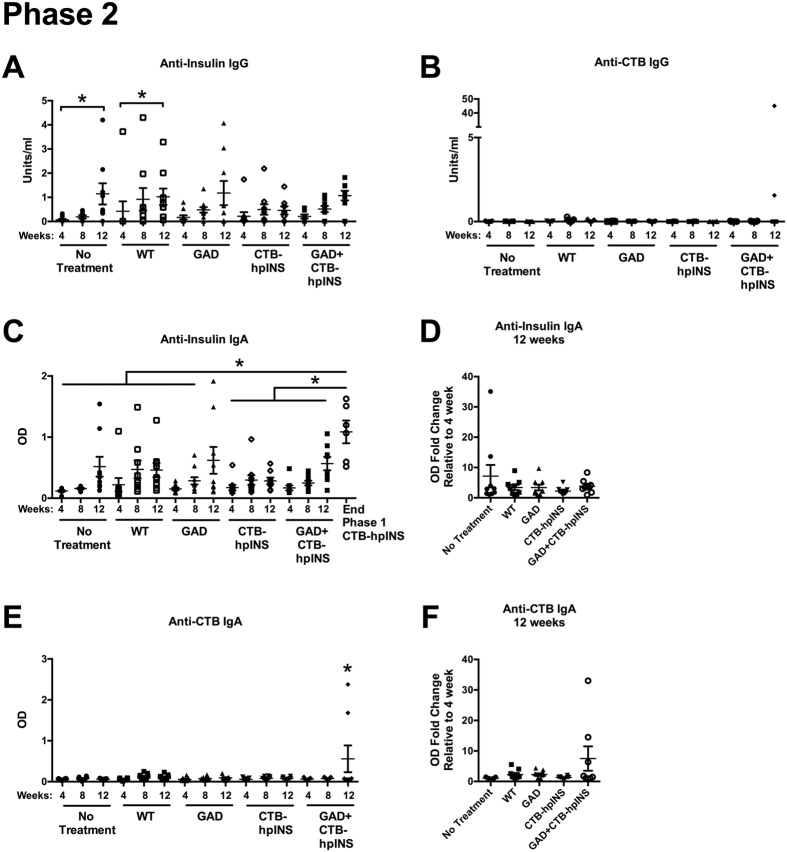
Phase 2: Serum (**A**) anti-insulin IgG, (**B**) anti-CTB IgG, (**C,D**) anti-insulin IgA, and (**E,F**) anti-CTB IgA were measured at 4, 8, and 12 weeks of age via ELISA. (**A**) Anti-insulin IgG was elevated in untreated and WT tobacco treated animals at 12 weeks of age relative to 4 weeks of age, *P* < 0.0001. (**B**) Anti-CTB IgG did not differ significantly across treatment groups over time, *P* = 0.37. (**C**) Serum anti-insulin IgA levels (as measured by absolute OD) were significantly higher in phase 1 CTB-hpINS treated mice (positive control) compared to all time points and treatment groups except for 12 week-old GAD tobacco treated animals, *P* < 0.0001, but (**D**) at 12 weeks of age, there was no difference between treatment groups as measured by OD fold change, *P* = 0.43. (**E**) At 12 weeks of age, anti-CTB IgA was elevated in mice that received combination therapy as measured by absolute OD, *P* < 0.01, but (**F**) the difference was not significant when measured by OD fold change at 12 weeks of age, *P* = 0.07 (ANOVA).

Anti-insulin and -CTB IgA levels were significantly increased at 12 weeks of age in mice treated with CTB-hpINS compared to WT tobacco (Fig. 6E,F). IgA class switching has been shown to occur in the presence of TGF-β^45^,^46^. Although this might suggest a tolerogenic effect, in those animals treated with CTB-hpINS tobacco, neither anti-insulin IgA (Fig. 6G) nor anti-CTB IgA (Fig. 6H) positivity predicted T1D onset. Anti-insulin IgG was also significantly elevated in CTB-hpINS-treated animals at 8 and 12 weeks of age (relative to 4 weeks) while titers did not increase significantly in WT-treated animals over time (Fig. 6I,J). However, titers were not higher in CTB-hpINS-treated animals compared directly against WT-treated mice at 12 weeks of age. Among CTB-hpINS-treated animals, insulin-specific IgG levels were not associated with eventual progression to T1D (Fig. 6K). During phase 2, however, the oral delivery of CTB-hpINS tobacco did not induce an insulin- or CTB-specific IgG or IgA response (Fig. 7). Serum collected from mice treated with CTB-hpINS during phase 1 was included as a positive control (Fig. 7C).

#### Oral Antigen Therapy Did Not Modulate the Immune Phenotype of the Insulitic Lesion

**Figure 8:**
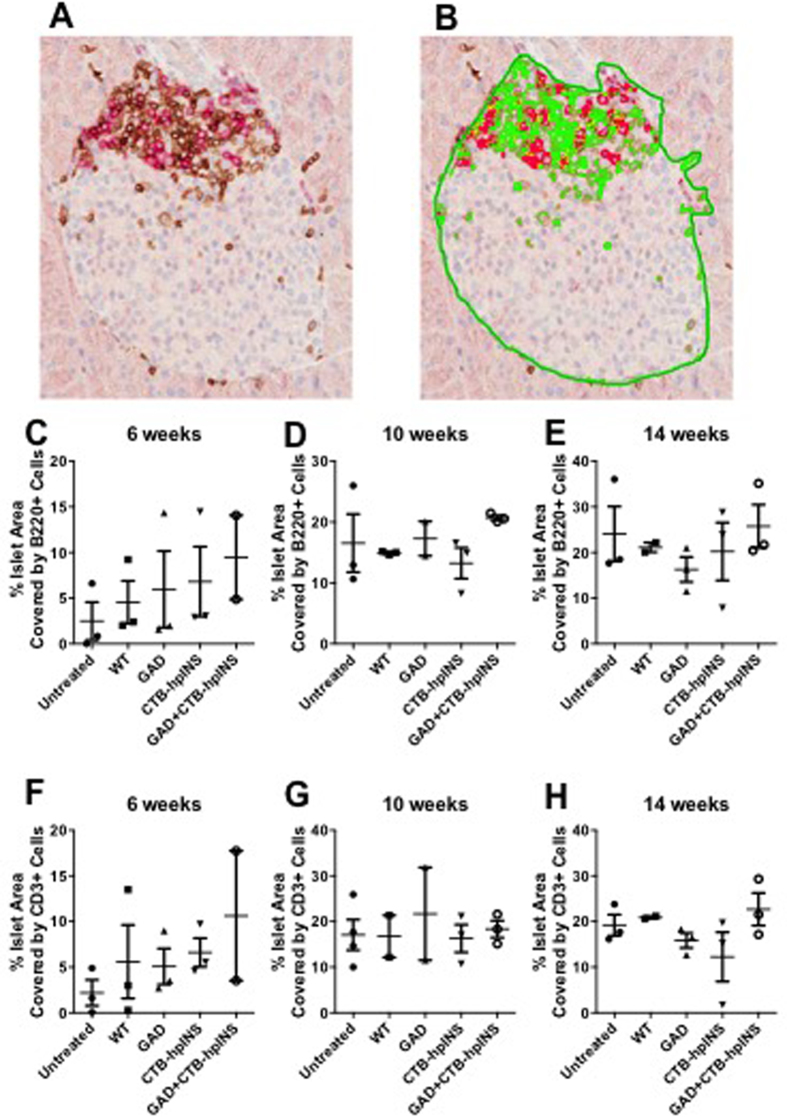
(**A**) Pancreas sections were stained for B220 (red) and CD3 (brown) via IHC. Annotations were drawn to include only islet area for analysis. (**B**) The percentage of islet area infiltrated by B220^+^ (red) and CD3^+^ (green) was quantified using an optimized algorithm. At (**C,F**) 6, (**D,G**) 10, and (**E,H**) 14 weeks of age there was no difference between treatment groups regarding (**C–E**) B220^+^ and (**F–H**) CD3^+^ islet area, *P* = ns, all (ANOVA). N = 2–3 mice per group per time point.

During phase 2, pancreata harvested at 6, 10, and 14 weeks of age were analyzed for CD3^+^ and B220^+^ cells within the islets. As expected, the percentage of islet area infiltrated by CD3^+^ and B220^+^ cells appeared to increase over time; however, for each time point, there were no significant differences between treatment groups (Fig. 8) suggesting that therapy did not reduce or delay T or B cell-mediated insulitis.

## Discussion/Conclusions

In this two-phase study, we sought to improve upon previous attempts to induce autoantigen-specific oral tolerance for the prevention of T1D by utilizing an innovative method for the mucosal delivery of two T1D-related autoantigens in a combination therapy. Transplastomic tobacco plants expressing GAD and CTB-hpINS were tested alone and in combination to optimize an oral tolerogenic vaccine for the prevention of T1D using the leading animal model of spontaneous autoimmune diabetes, the NOD mouse. We hypothesized that oral therapy would not only result in synergism but also, overcome limitations associated with oral therapy - namely, autoantigen digestion - affording efficient autoantigen delivery for improved efficacy. Furthermore, we expected that co-administration of the tolerogenic adjuvant, ATRA, would promote tolerance induction to elicit the desired immunological effect in NOD mice, a strain that reportedly exhibits impaired mucosal tolerance mechanisms relative to non-diabetes-prone strains^47^,^48^.

In this study dosage is referred to the amount autoantigen gavaged in NOD mice, but dose delivered to circulation or the immune system was not quantifiable. The observed suboptimal outcome with monotherapies (phase 1) left room for us to test for synergism in combination treatment and suggests that a short-course of treatment may not be sufficient for lasting tolerance induction, which is in agreement with previous reports using plant cells expressing FVIII and FIX to induce oral tolerance^24^,^25^. Expressed another way, we hypothesized that the tolerogenic effect of therapy was lost upon the cessation of treatment in phase 1 and that continued oral therapy would result in extended protection from T1D onset. Thus, the medium dose of CTB-hpINS and the highest dose of GAD were selected for a combination therapy during phase 2. Unfortunately, these protocol modifications did not result in improvements in terms of disease prevention. Kaplan-Meier survival curves for each tobacco treatment were comparable between phases 1 and 2, suggesting that initial stopping of therapy did not precipitate T1D onset and that ongoing treatment in phase 2 did not improve efficacy. Oral autoantigen therapy alone may thus, not be successful in preventing T1D, regardless of treatment duration. Moving forward, it may be beneficial to consider a combination of two individual treatments that demonstrate some, though limited success, as shown by Bressen *et al*.^49^.

With respect to other observations of potential importance, oral tobacco therapy (including WT) resulted in reduced weight gain in young mice compared to untreated animals. Hence, it is possible that the metabolic stimulus of nicotine or reduced food consumption may have been attributable to gavage of large quantities of plant cells; nonetheless, the metabolic effects of treatment may have influenced disease kinetics. Oral therapy with plant cells expressing CTB-hpINS or GAD appeared to delay T1D onset in both phase 1 and phase 2 studies in NOD mice, but this effect was not statistically significant or sustained with prolonged treatment. Beyond this, while our cohorts were not undersized by current standards, without greater statistical power it cannot be definitively determined whether small differences (22% in phase 2) in T1D incidence were attributable to autoantigen therapy or unforeseen effects of oral tobacco treatment. This argues that the pressure to use minimal animal numbers may not be suitable when evaluating combination therapies for disease prevention in NOD mice where colony- and batch-specific effects may influence T1D kinetics.

We anticipated oral therapy would induce antigen specific tolerance and potentially, infectious tolerance. Instead, splenocytes from CTB-hpINS treated animals showed reduced production of IL-10 production under GAD stimulation compared to untreated controls signifying a potential off-target effect of the therapy that none-the-less, failed to induce bystander suppression capable of preventing disease. In most previous studies using antigens bioencapsulated in plant cells for the induction of oral tolerance, IL-10 and/or Treg induction was routinely observed^3^,^22^,^24^,^25^,^26^,^29^. Thus, our findings may be related to the known T1D-related defects in Treg function^50^ and indicate a need for a more aggressive approach to tolerogenic therapy. Alternatively, this could be the result of insufficient delivery of GAD to the mucosal immune system since this protein could not be expressed in tobacco as a fusion protein with CTB. Oral delivery of plant leaves expressing autoantigens also failed to modulate the frequency of CD4^+^CD25^+^Foxp3^+^ Tregs or LAP^+^ T cells in MLN and PLN. This said, future investigations should include flow cytometric analysis of cytokine expression and Th signature transcription factors among insulin-tetramer^+^ T cells in each of these tissues to more conclusively determine the antigen-specific effects of oral therapy.

Interestingly in phase 1, oral CTB-hpINS treatment resulted in increased levels of insulin- and CTB-specific IgA and IgG, but neither was associated with eventual progression to T1D. Oral tolerance has been shown to reduce antigen-specific IgA and IgG in response to oral challenge^51^, hence our findings may indicate therapy failure which aligns with the observed therapeutic outcome. Additionally, it is possible that CTB- and/or insulin- specific IgA could reduce bioavailability of the fusion protein at the mucosal surface, thereby limiting therapeutic effect over time. Thus, further investigation is needed to discern the significance of these humoral immune responses observed during phase 1. Serum collected at T1D onset or 32 weeks of age did not contain elevated anti-insulin or -CTB IgG and IgA, and mice from phase 2 efforts did not seroconvert at any point during the course of treatment. This discordance is certainly intriguing as the only difference in vaccine formulation was the exclusion of tocopherol-stripped corn oil during the second phase of the study. Indeed, this is most likely due to adjuvant effect of corn oil as shown in a recent study in which plant oil compounds increased polio antigen antibodies (IgA, IgG1)^52^. It is possible that the oil, which is largely composed of linoleic acid, may have directly exerted unforeseen immunological effects^53^,^54^,^55^,^56^ or alternatively, might have affected antigen or immune cell trafficking in the lymph^57^,^58^.

Together, our data suggest that weekly oral autoantigen therapy, alone, is not sufficient to prevent spontaneous T1D in the NOD mouse, and that the delivery of two autoantigens in combination does not result in synergism, possibly because GAD was not fused with CTB. This result was quite surprising given comparison to previous accounts of oral tolerance induction in NOD mice^8^,^9^,^10^,^16^,^17^,^18^,^19^,^59^. However, we do find it corroborative of somewhat less-discussed notions that mucosal tolerance mechanisms are likely confounded in NOD mice and autoimmune-prone individuals^15^,^47^,^48^,^60^,^61^, even in instances of nasal immunization with foreign antigen, such as hen egg lysozyme or ovalbumin^47^,^48^. In that regard, inclusion of some form of “positive control” would have clearly benefitted these studies and if identified, should be included in future efforts. This said, previous studies using transplastomically expressed antigens for oral tolerance have demonstrated robust efficacy in preventing the development of anaphylactic response to peripherally administered antigen^24^,^25^,^26^ suggesting that the inability to prevent autoimmunity and T1D may stem from the disease and its animal model (e.g., self antigen vs. foreign, autoimmune NOD vs. immunologically “normal” mice) rather than the delivery vehicle.

A critical evaluation of the early literature would reveal that in studies involving multiple NOD colonies, T1D incidence in NOD mice was *incredibly* low even among control animals^8^,^10^. For example, “prevention” in one study saw a disease frequency of approximately 48 percent in control female NOD mice, versus 30 percent of oral insulin treated mice, at one year. Beyond this, other studies yielded inconsistent outcomes regarding the most effective form of antigen, combinatorial agents, and treatment regimen^10^,^16^,^17^,^18^,^59^,^62^. In fact, a recent effort by Pham *et al*. demonstrated that oral therapy with unprotected insulin protein does not prevent T1D in NOD mice suggesting a need for autoantigen encapsulation^15^. Here we encapsulated two autoantigens (hpINS and GAD) using a plant-based expression system yet were unable to induce oral tolerance or significantly prevent disease.

However, in no way does this unequivocally discount antigen-specific immunotherapy as a means for intervention but rather, calls for further treatment optimization. For example, low dose anti-CD3 plus oral inoculation with *Lactococcus lactis* expressing IL-10 and either GAD or hpINS has demonstrated remarkable efficacy toward the reversal of new-onset T1D in NOD mice^35^,^63^. Plant-based autoantigen delivery provides the benefits of more accurate dosing and reduced complications associated with maintaining live bacterial cultures for clinical translation. Therapeutic proteins are expressed at exceptionally high levels, and lyophilized plant cells can be stored for several months or years without a decrease in their functionality, thereby eliminating prohibitively expensive processes currently required for the production of protein drugs including fermentation, purification, cold storage/transportation and sterile delivery^64^,^65^. Thus, we anticipate that a similar approach using potent immune-depleting agents (e.g., Thymoglobulin, anti-CD3) prior to oral treatment and tolerizing agents (e.g., GCSF, IL-10) in combination with the transplastomic plant cells will build upon these findings and potentially provide a therapy more suitable for clinical use.

## Additional Information

**How to cite this article**: Posgai, A. L. *et al*. Plant-based vaccines for oral delivery of type 1 diabetes-related autoantigens: Evaluating oral tolerance mechanisms and disease prevention in NOD mice. *Sci. Rep.* **7**, 42372; doi: 10.1038/srep42372 (2017).

**Publisher's note:** Springer Nature remains neutral with regard to jurisdictional claims in published maps and institutional affiliations.

## Supplementary Material

Supplementary Figures and Legends
