# Supplementary material for: Plant-based vaccines for oral delivery of type 1 diabetes-related autoantigens: Evaluating oral tolerance mechanisms and disease prevention in NOD mice
Source: Sci Rep. 2017 Feb 13;7:42372. doi: 10.1038/srep42372 (PMC5304332; doi:10.1038/srep42372)
Supplement: Supplementary Figures and Legends [file srep42372-s1.doc]

**Plant-based vaccines for oral delivery of type 1 diabetes-related autoantigens: Evaluating oral tolerance mechanisms and disease prevention in NOD mice**

Amanda L. Posgai, Clive H. Wasserfall, Kwang-Chul Kwon, Henry Daniell, Desmond A. Schatz, Mark A. Atkinson

**Supplemental Fig. S1.**


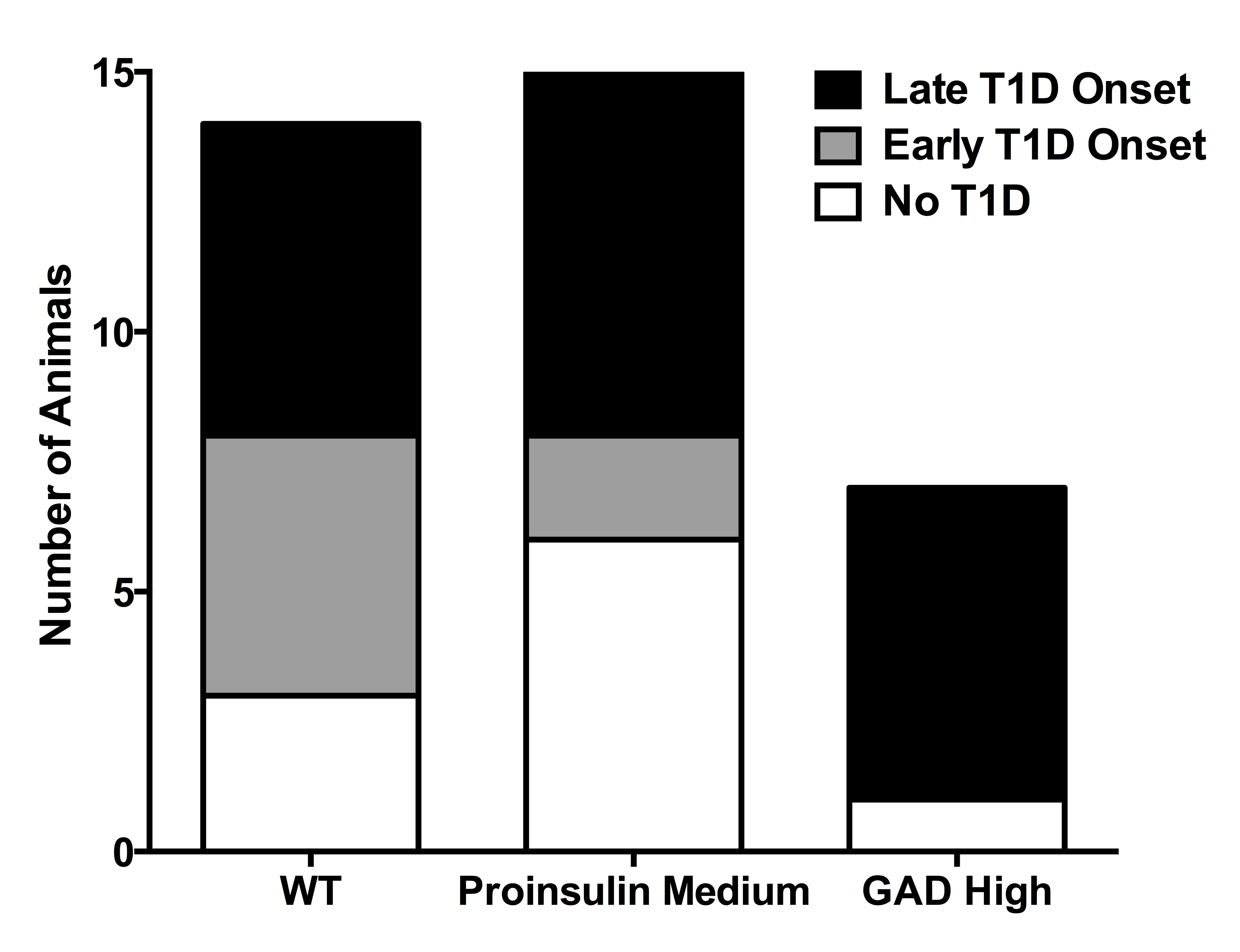


Mice that received oral treatment with 250µg CTB-hpINS (Proinsulin Medium) and 500µg GAD (GAD High) demonstrated a trend toward reduced frequency of early Type 1 Diabetes (T1D) onset compared to wild type tobacco (WT)-treated animals, *P* = 0.07 (Chi-square test).

**Supplemental Fig. S2.**

**
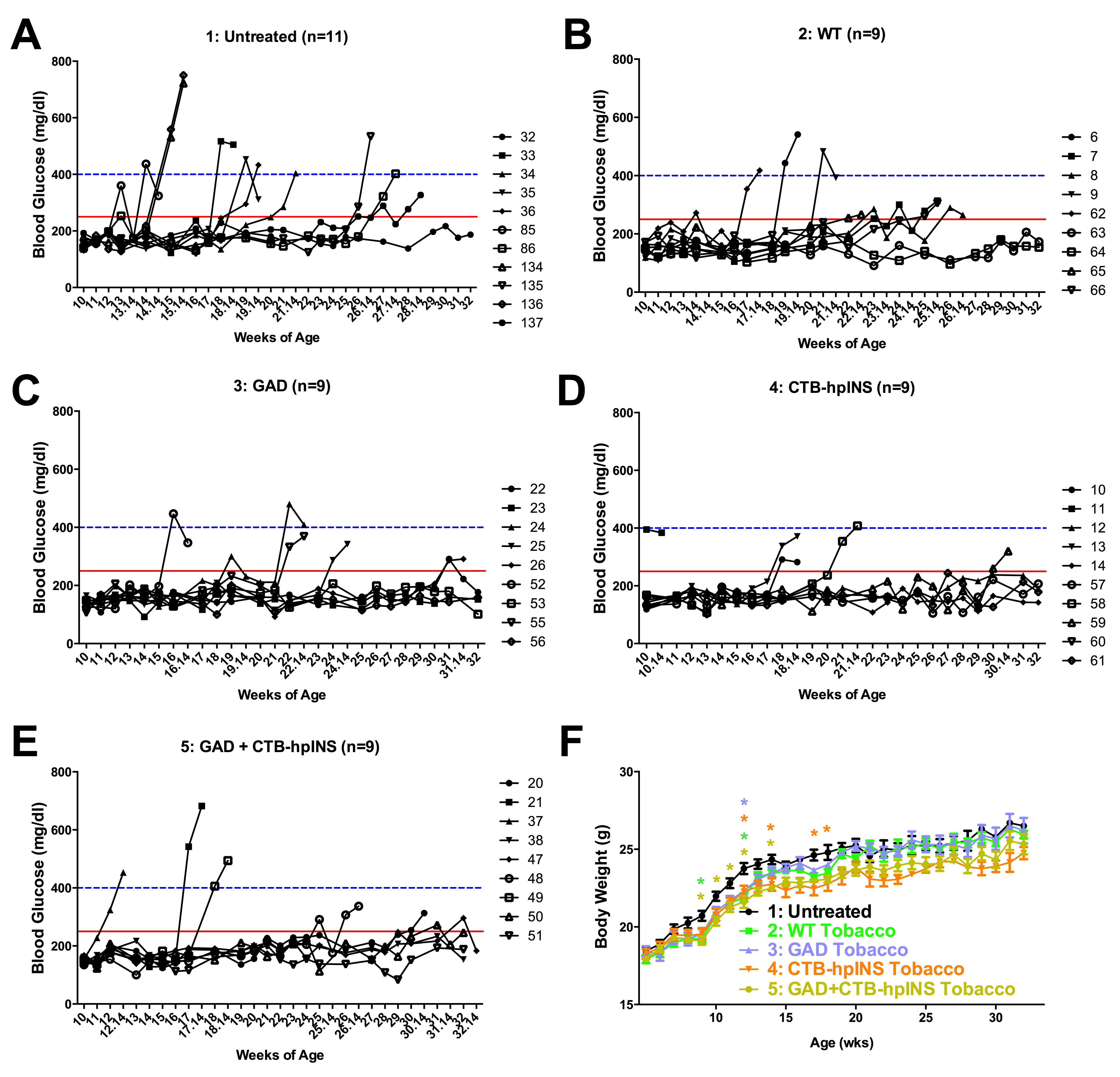
**

Blood glucose values (mg/dL) over time are shown for untreated (A), WT-treated (B), GAD-treated (C), CTB-hpINS-treated (D), and combination-treated (E) animals. Number of animals (n) per group is indicated in the figure. Body weight (g) is plotted across the lifetime of the animals beginning at 5 weeks of age (F). Oral therapy, including WT control, reduced weight gain in young NOD mice compared to untreated animals at various time points, but by the end of the study, there was no significant difference between treatment groups, * *P* < 0.05 (Two-way ANOVA). Significance for WT (green), GAD (blue), CTB-hpINS (orange), and combination therapy (gold) is represented by * color at a given time point, relative to untreated animals (black). Data are represented as mean ± SEM at each time point for each treatment group.

**Supplemental Fig. S3.**


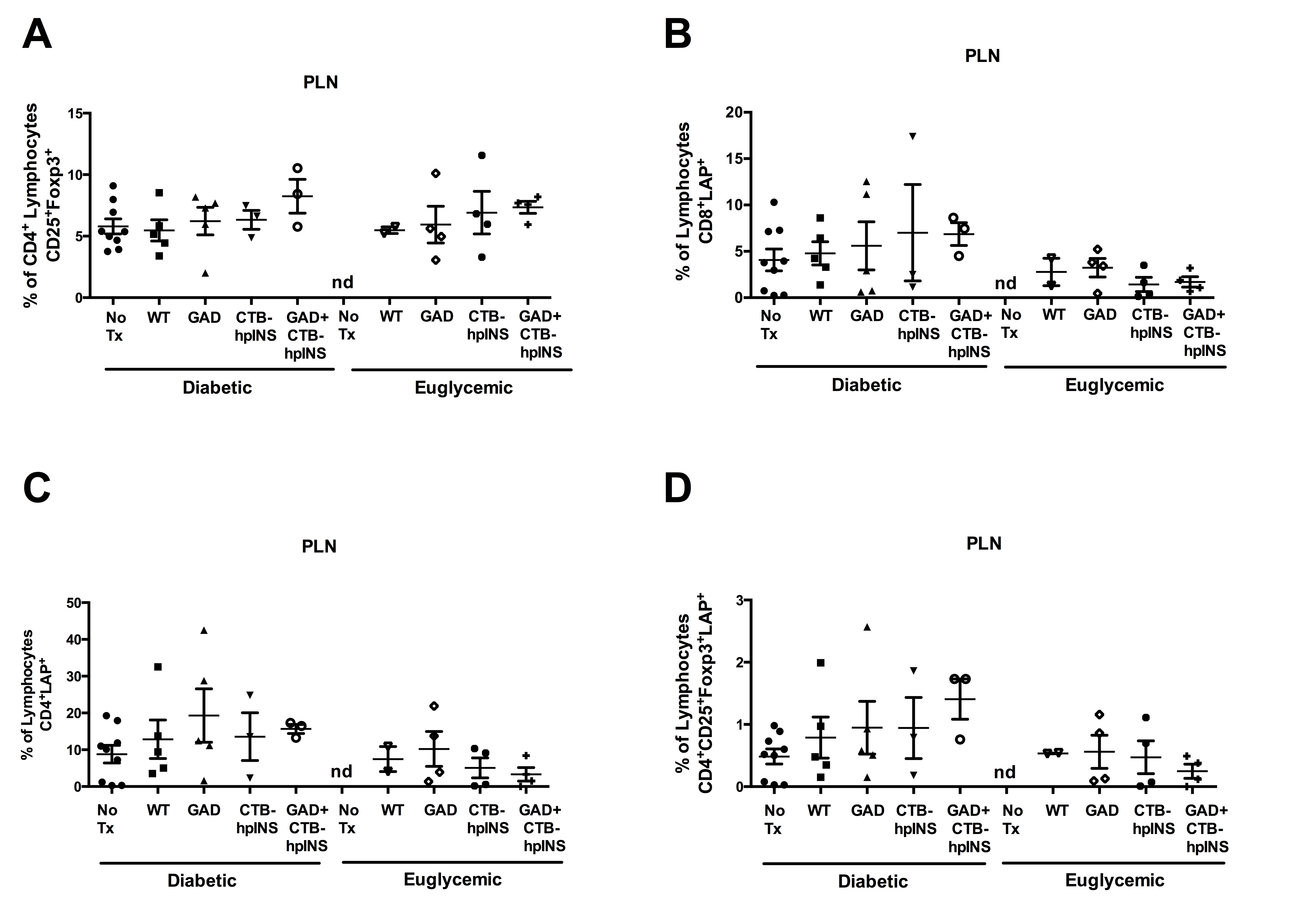


At T1D onset or 32 weeks of age, fresh PLN cells were stained for CD4, CD8, CD25, LAP, and Foxp3 for flow cytometric analysis. Lymphocytes were gated on forward and side scatter. Dead lymphocytes staining strongly positive for LIVE/DEAD® Fixable Near-IR were excluded. Within the PLN, the frequency of CD4^+^CD25^+^Foxp3^+^ Tregs (A), CD8^+^LAP^+^ T cells (B), CD4^+^LAP^+^ T cells (C), and CD4^+^CD25^+^Foxp3^+^LAP^+^ Tregs (D) did not differ between diabetic and euglycemic animals, *P* = 0.48, *P* = 0.31, and *P* = 0.31, respectively.

**Supplemental Fig. S4.**


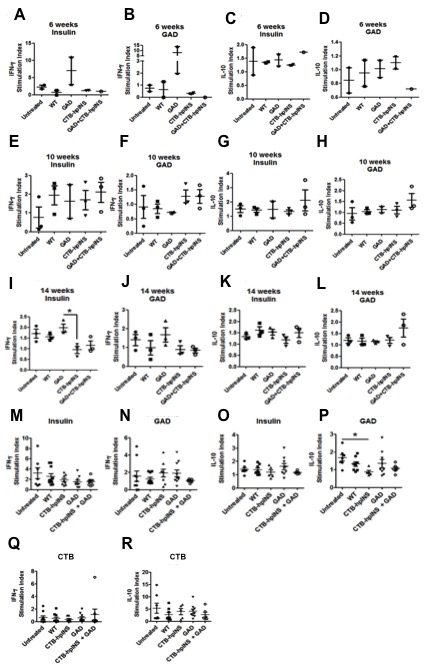


At (A-D) 6, (E-H) 10, and (I-L) 14 weeks of age as well as at (M-R) longitudinal time points (T1D onset or 32 weeks of age), fresh splenocytes were stimulated with (A, C, E, G, I, K, M, O) recombinant human insulin, (B, D, F, H, J, L, N, P) GAD65, or (Q-R) CTB and analyzed for (A, B, E, F, I, J, M, N, Q) IFN-γ or (C, D, G, H, K, L, O, P, R) IL-10 production via ELISPOT assay; N = 1-3 mice per group at cross-sectional time points and N = 9-11 per group at longitudinal time points as indicated by number of data points in each graph. At 6 weeks of age, statistical analyses were not possible with N<3 for all groups (A-D). No significant differences were observed at 10 weeks of age (E-H), *P* = ns, all. (I) At 14 weeks of age, insulin stimulated IFN-γ production was reduced in CTB-hpINS treated animals relative to those treated with GAD, *P* < 0.05. (J) At 14 weeks, GAD stimulated IFN-γ production did not differ across treatment groups, and (K, L) there were no differences in in IL-10 production regardless of the stimulatory conditions, *P* = ns, all. At longitudinal endpoints, treatment had no effect on (M, N, Q) IFN-γ production, regardless of the stimulation condition and on IL-10 production with (O) insulin or (R) CTB stimulation, *P* = ns, all. (P) Under GAD stimulating conditions, treatment with CTB-hpINS tobacco was associated with reduced IL-10 production compared to untreated control animals (Kruskall-Wallis test).

**Supplemental Fig. S5.**


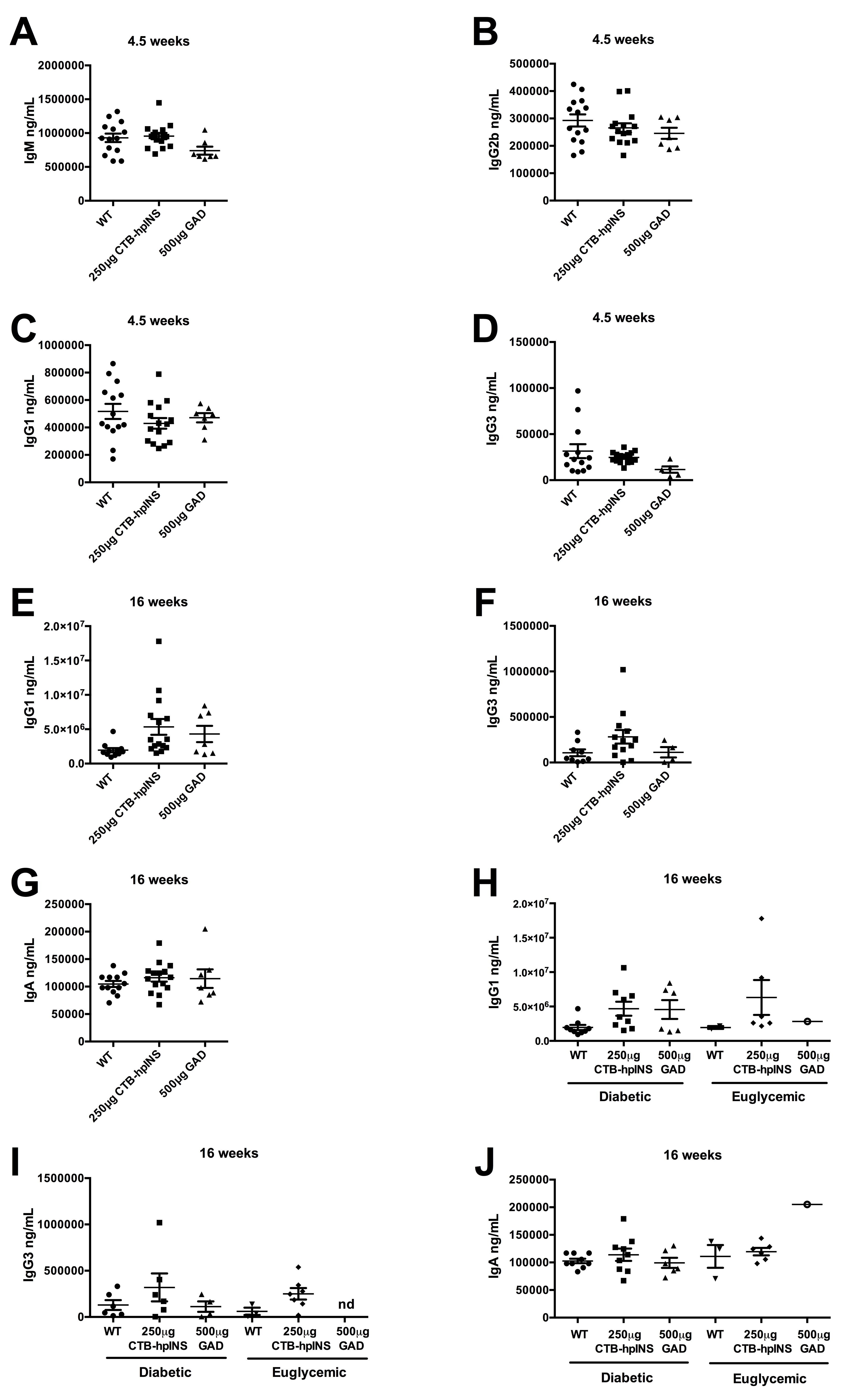


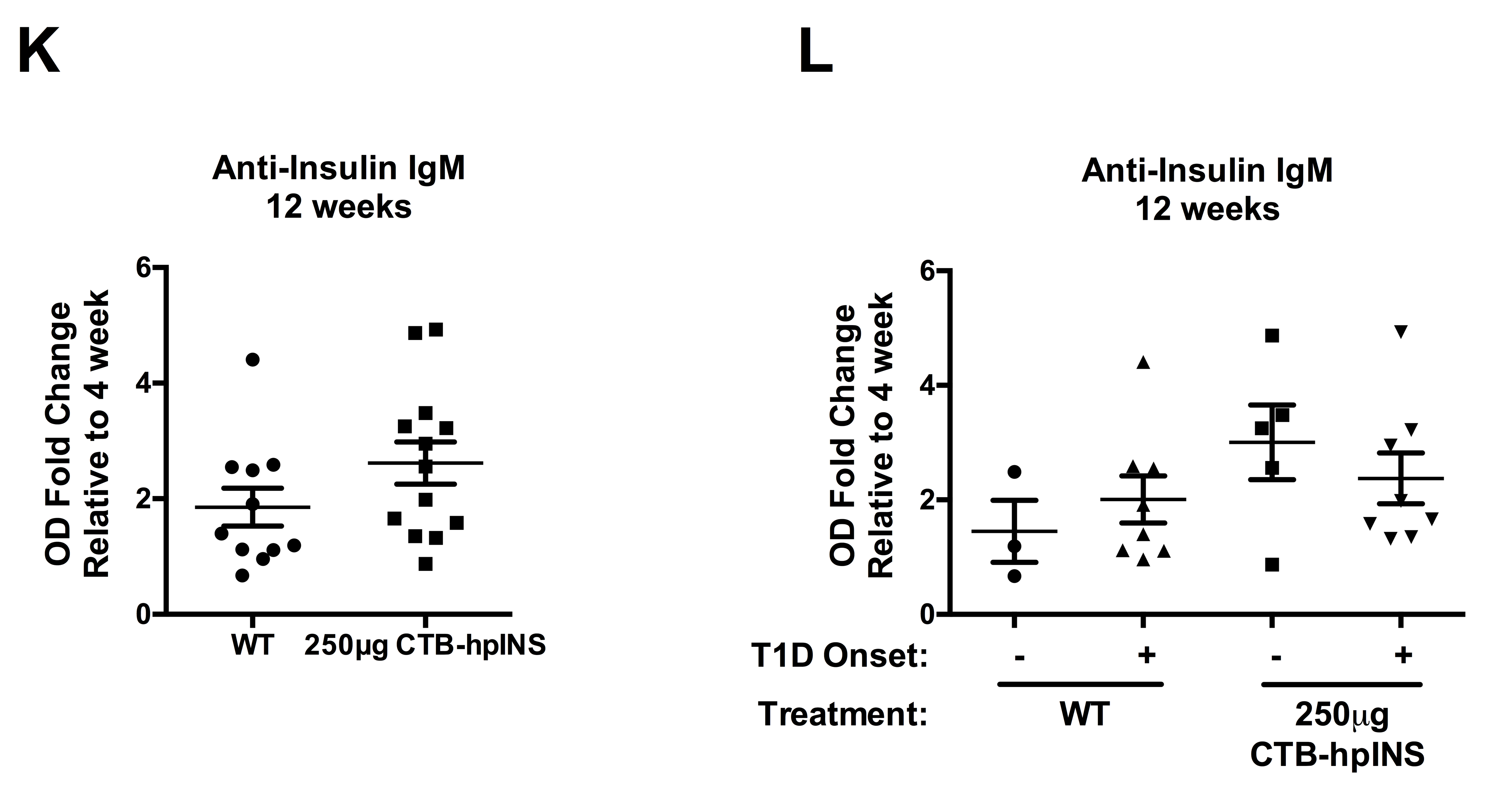


Prior to the initiation of oral tobacco treatment, at 4.5 weeks of age, there was no difference between treatment groups in total serum IgM (A), IgG2b (B), IgG1 (C), or IgG3 (D) as measured by luminex assay, *P* = ns (ANOVA). At 16 weeks of age, total serum IgG1 (E, H), IgG3 (F, I), and IgA (G, J) did not differ across treatment groups at 16 weeks of age for animals that would remain euglycemic or progress to hyperglycemia, *P* = ns (all, ANOVA).
